# Static vs. dynamic methods of delivery for science communication: A critical analysis of user engagement with science on social media

**DOI:** 10.1371/journal.pone.0248507

**Published:** 2021-03-31

**Authors:** Sarah A. Habibi, Lidya Salim

**Affiliations:** 1 Faculty of Science, Ontario Tech University, Oshawa, Ontario, Canada; 2 Applied Bioscience Graduate Program, Faculty of Science, Ontario Tech University, Oshawa, Ontario, Canada; University of Pisa, ITALY

## Abstract

Science communication has been increasingly viewed as a necessity and obligation of scientists in recent years. The rise of Web 2.0 technologies, such as social media, has made communication of science to the public more accessible as a whole. While one of the primary goals of science communication is to increase public engagement, there is very little research to show the type of communication that fosters the highest levels of engagement. Here we evaluate two social medial platforms, Instagram and TikTok, and assess the type of educational science content (ESC) that promotes user awareness and overall engagement. Specifically, we measured the level of engagement between static and dynamic posts on Instagram, and lecture-style and experimental videos on TikTok. User engagement is measured through the analysis of relative number of likes, comments, shares, saves, and views of each post in the various categories. We found that users interact with ESC significantly more (p<0.05) when the content is presented in dynamic ways with a component of experimentation. Together, we took the findings of this study and provided a series of suggestions for conducting science communication on social media, and the type of ESC that should be used to promote better user outcomes.

## Introduction

Educators have been studying the best pedagogical practices for increasing student engagement in science classrooms for decades. Considering the rate at which technology and society are changing, educators must be mindful of how students learn as a result of these changes. Science classrooms are shifting further away from the traditional lecture style and more towards videos, experimentation, and discovery. Some could argue that one of the main driving factors in this rapid change has been the introduction of Web 2.0 technologies, including social media [1]. The way in which social media has impacted how students learn and engage with society is evident [2]. As a result, science educators have been incorporating more online learning and forms of technology and social media into their classrooms and teaching [3–5]. At the same time, scientists, teachers, and other experts have begun using social media as a medium to stimulate discussion of science beyond the classroom [6]. The practice of engaging the public into science and stimulating discussion and discourse of scientific topics is referred to as science communication (SciComm). More specifically, the contemporary definition of SciComm is “the use of appropriate skills, media, activities, and dialogue to produce one or more of the following personal responses to science: awareness, enjoyment, interest, opinion-forming, and understanding” [7].

In recent years, much focus has been put on social platforms such as Twitter, Facebook, LinkedIn, and Instagram for increasing societal engagement with scientific information [8, 9]. Since public engagement with science is accepted as a necessity, and as a duty of scientists [10, 11], many have taken their SciComm online. As a result, a whole community of “science communicators” was formed. With the influx of scientific information being communicated on social platforms, one must always question whether all the information posted is accurate, reaches the appropriate audience, and is effective at fostering user engagement. Although it is well understood that social media increases student engagement as a whole [3], very little is known about the best methods for SciComm on social media, let alone the type of educational science content (ESC), that receives the most engagement and discourse on the various platforms.

The present study looks at the prevalence of user engagement with ESC on two major social media platforms, Instagram and TikTok. We highlight the type of ESC that users are more likely to engage with and share, and how that differs between different social platforms. We also evaluate the type of ESC that results in the highest retention of user.

### Background

#### Engagement of non-scientists in science

There has been a growing concern regarding the need for scientists to engage in scientific discussions with society, in hopes to create a more scientifically literate world [10]. Of even more importance is the need for engaging not only adults, but also youth, in these discussions [12]. The term “engagement” is often used when describing the practice of sharing and receiving information with the public in a two-directional way. While promoting discussion among individuals does foster engagement, some suggest additional practices are required to obtain full engagement. Row and Frewer (2005) describe public engagement as the combination of three things, public communication, public consultation, and public participation. In recent years, it has generally been established that not only is it important to engage the public in scientific discussion, but it is also necessary to engage scientists in having those discussions [11, 13, 14]. As a result, much research now focuses on how scientists perceive SciComm as a duty, and little comes from how it should be practiced to ensure efforts are effective.

The growing technological-driven society and prevalence of social media in our daily lives has begun reshaping how the public engages with science and vice versa. With traditional news outlets, such as newspapers and televised programming, becoming increasingly less popular, people have shifted to alternative media, such as the internet and social media to obtain science news [15]. It has been shown that SciComm on social media platforms such as Twitter and Facebook acts to increase user engagement and discussions in science-related conversation [8, 16, 17]. Similarly, engagement of the public into scientific conversations is not limited to specific subject areas, with positive experiences being reported in broad areas ranging from the plant sciences all the way to earth and climate sciences [9, 17]. With many scientists viewing engagement as a way to recruit future scientists [18, 19], an increasing number of research groups are using social media as a recruitment tool [20]. More recently, social media has also been used to engage the public into community science (formerly referred to as citizen science) [21, 22], a practice where scientists call on volunteers from the public to take part in local research initiatives. Overall the multi-dimensional use of social media clearly plays a significant role in engaging the public into science.

While it is evident that scientist have begun prioritizing engagement of the public into scientific discussion, very little research has identified the best practices for such SciComm, and the type of ESC that maximizes user engagement. Research highlights the need for communication trainers to help scientists formulate their SciComm initiatives [11]. This formal SciComm training is also not limited, with many suggesting that undergraduate students, graduate students, and faculty could all benefit from it [12, 13]. Coupled with the need for including youth in scientific discussions, our group aims to address the type of SciComm and ESC that fosters better user engagement and promotes discussions among the future generation of scientists.

#### Social media as a medium for science communication

Within the last decade, social media has become integrated into the lives of so many people all over the world. With applications like Instagram, Facebook, and Twitter, it has become increasingly easier to share photos, videos, news, and a variety of information with people in many geographical locations. As of 2020, it has been reported that over 200 million individuals in the United States are active on social media platforms (Statista), accounting for over 75% of the total population. The prevalence of social media in society is evident and its growth as we move into the future is inevitable. Social media provides the optimal medium for information sharing, the creation of community, engagement of groups of people, and a place for interactivity. For these reasons, experts from many professional backgrounds suggest the importance of using social media as a tool for sharing educational content online [3, 20]. It has been stressed that “adequate communication involves information the recipient needs, access to that information, and in a form they can comprehend” [23], all of which is easily achieved through social media.

Instagram, released in 2010, is a social networking app that encourages its users to share photos and videos with one another and engage with communities of similar individuals. The majority of Instagram users are between the ages of 18–34 (Statista), highlighting its prevalence among the younger generation. While Instagram originally was dominated by fashion bloggers and “beauty gurus”, in recent years more businesses and professionals have been popping up on the app. In particular, the scientific community on Instagram has blossomed, with scientists speaking on a variety of topics including astrophysics, neurodegenerative diseases, climate change and weather, and much more. Social media provides a place for scientific discussions to be had at accelerated rates, regardless of geographical location, with both scientist and non-scientists [20]. Similarly, with research indicating how SciComm in the form of self-portraits on Instagram can reduce the negative attitude society has towards scientists [6], it is evident that the practice of SciComm on social media has much potential. That being said, although there is an overflow of science content being posted on Instagram, very little is understood about the type of content that fosters user engagement. It is one thing to present and teach scientific information to society, it is another thing to ensure that people are engaged with what is being taught. In 2016, Instagram introduced a business feature onto the platform, which included the collection of analytical data called “Insights”. Data collected from each Instagram account includes the number of people viewing the account, how many times posts have been seen, liked, shared, and saved, and the demographic information of the accounts’ users (age, gender, location). Although the Insights feature was introduced to support businesses to aid in understanding their consumers, the analytical data also provides an opportunity for content creators to learn about the type of content their users connect with most. From an educator standpoint, we see how this data can drive research into understanding the best methods of delivery for SciComm on social media.

Like many forms of technology, social media is constantly growing and changing to better suit the needs and wants of its users. As a result, new social networking apps such as TikTok are pulling up a seat to the table. TikTok is a video sharing app that encourages its uses to use musical backgrounds or voiceovers as the basis for the videos. Since TikTok was released in 2017, its popularity has been growing exponentially. TikTok’s analytics company, Sensor Tower, released in May 2020 that the app had over 315 million downloads since January 2020, making it the single largest number of downloads in one quarter. This could possibly be in response to the global COVID-19 pandemic, which caused millions of people to work from home and primarily use technology for entertainment. In response to this, in April 2020 TikTok pledged “$250M to support front line medical workers, educators, and local communities deeply affected by the global crisis” (TikTok Newsroom, 2020). One of these efforts was the Creative Learning Fund, which provided “$50M in grants to educators, professional experts, and nonprofits whose real-world skills and expertise can help spread educational information and useful course material in an accessible, ‘distance learning format’”. With such a large audience of young science learners online, our team saw this as a great opportunity to assess how users engage and learn from ESC on social media. Similar to Instagram, TikTok has an analytics feature for its users to track data from their videos. Data collected includes the number of video likes, comments, shares, total play-time, total views, average watch time of videos, and audience demographics.

#### The importance of experimentation in science education

Growing scientific evidence shows that hands-on experimentation with science is one of the most effective ways for students to learn, engage with, and retain scientific information and skills [5, 24–26]. Similarly, the introduction of active hands-on science experimentation into classroom teaching, produces students with a more positive attitude towards the sciences [27]. This could in part be due to the greater amount of interest the students have in taught material when there is a component of experimentation and investigation involved [28].

In response to research that displays the importance of experimentation in the sciences, and an understanding that not all students and schools have access to the means necessary to conduct such experiments, “mobile” science laboratories have become increasingly more popular [5]. In fact, a recent 2017 survey highlighted that over 1.2 million students across the United States have taken part in some form of a mobile science lab [5]. Similarly, organizations such as Science Rendezvous have begun to grow and develop, as the need for science experimentation and engagement of the public into scientific conversation increases. In 2019, over 215,000 attendees participated in the 1000+ unique hands-on science experiments that Science Rendezvous offers, a new milestone for such events in Canada (Science Rendezvous, 2020).

While it is evident that people of all ages enjoy taking part in science experimentation, we have to remember that even mobile labs and science outreach may not be accessible to everyone. At the height of the 2020 global COVID-19 pandemic, the idea that in-person experimentation may not be an option became a reality for many North Americans. As a result, many educators, scientists, and professionals had to adapt to the “new normal” and take their work home. Social media provides the optimal medium for the continuation of classroom learning and scientific discussions, as it does not depend on the geographical location of the scientist or the people who receive and engage with the information.

### The current study

The present study aims to assess the type of educational science content (ESC) that fosters user engagement on social media, and how it differs between platforms. The two social media platforms that we analyze here are Instagram and TikTok. We focused our study on these two platforms for three reasons. First, Instagram and TikTok are primarily photo and video sharing applications, so they provide an optimal platform for sharing the type of ESC used in this study. Second, this study aimed to target and engage a younger audience, as they may comprise the next generation of scientists. The primary audience on Instagram and TikTok are of a younger generation, with the largest number of users being between the ages of 18–34 and 10–29, respectively (Statista). In comparison, other popular platforms like Twitter and Facebook tend to engage an older population, with the largest number of users being between 25–49 and 25–45, respectively (Statista). Third, to the best of our knowledge, there are few reports regarding user engagement with SciComm efforts on Instagram as well as no reports involving TikTok. The literature presented showcases the lack of quantitative data available for assessing the levels of public engagement when viewing ESC on social media. Similarly, methods for measuring user engagement directly on social platforms have yet to be studied. As a result, we have conducted a critical analysis of the analytical data collected on Instagram and TikTok and formulated a method for accurately assessing what each piece of analytical data means in relation to user engagement. Both Instagram and TikTok record the number of likes, comments, views, and shares of every video posted. We have defined each of these four variables in relation to user engagement. As reported, true user engagement is achieved when users are communicating, consulting, and participating with the information being presented [29]. With this in mind, we ranked each of the four variables in order from lowest to highest levels of engagement: likes, views, comments, and shares.

The notion of “likes” on social media is viewed as a form of social currency, which relates to ones acceptance of the information they are receiving [30]. In relation to engagement, we define likes as a person acknowledging that the information is “good”, and overall enjoying it. When an individual “likes” content, they are showing interest in that information. Since it has been shown that an individual showing “interest” in science is significant for sustaining engagement [31], we suggest that the notion of liking ESC on social media is an important indicator of user engagement. As the number of likes on a post go up, the visibility of that post to a broader audience also increases, thus allowing more individuals an opportunity to engage with the information. The total number of times a post has been seen can be reflected in the “views”. We rank this level of engagement as higher because it captures how many times an individual re-watches the information presented. The act of watching a video multiple times attributes to an individual dedicating additional time to the information. Engagement in the form of views is important for ESC because it means that users are taking the time to review and learn more about the science presented. The next level of engagement is achieved through “comments”. Comments allow users to share their thoughts, ideas, concerns, appreciation, approval, or disapproval for the information shared. Regardless of whether or not the user is accepting of the ESC, it provides an opportunity for scientists to engage in discussions with the public and address concerns. This helps to reduce the spread of misinformation and allows for fast-paced discourses between scientists and society. The highest level of engagement is achieved when users “share” the information presented. The ultimate goal is to promote the spread of accurate scientific information among member of the public through engagement with scientist [13]. The act of a user sharing ESC with their friends and family helps to achieve this goal, by allowing a space for additional discussions to be had.

The ESC presented on Instagram takes the form of either static photos or dynamic videos. Whereas the ESC on TikTok falls within three categories, long descriptive videos, quick facts, and science experiments. Engagement with ESC on each platform will be assessed using the four analytical outputs defined above. To carry out this study, one of the researchers of this paper took part in the TikTok Creative Learning Fund as an educator, and discussed various scientific concepts (in the form of ESC) using these methods of delivery.

### Research question and hypotheses

Based on the literature that shows experimentation increases engagement with science, in addition to the prevalence of social media in our daily lives and the use of it as a tool for engaging the public into scientific discussion, we predict that:

**H1a:** Users engage with dynamic experiments more than they do with static images.

**H1b:** Users share dynamic educational science content (ESC) more than they share written ESC.

**H1c:** Users engage with fast-paced ESC more than they engage with long ESC videos.

In doing so, we are interested in addressing the following research questions:

**RQ1:** Do users on social media (TikTok and Instagram) engage with science experiments and information more when the content is presented in dynamic ways?

**RQ2:** What type of ESC are users more likely to share and engage in discussions with on various social platforms?

**RQ3:** Which social media platforms receives the highest amount of reach per post?

## Materials and methods

### Data collection on social media platforms

The analytical data assessed in this study was collected in accordance with the terms and conditions of TikTok and Instagram. The Research Ethics Board at Ontario Tech University (REB #16143) approved the secondary use of data obtained for analysis purposes.

The TikTok Creative Learning Fund program ran for six consecutive weeks between May–June of 2020. A total of 40 videos were posted (5 videos/week) during this time. The videos posted are representative of educational science content (ESC). The three categories of videos were, 40–60 second long lecture-style (primarily speaking) videos, 15 second short lecture-style (quick facts) videos, and 40–60 second long experimentation videos. Analytical data from each post was collected 2 weeks after the conclusion of the Creative Learning Fund program. Data included number of likes, views, comments, shares, length of video, and average watch time.

ESC was posted to Instagram over the duration of 1 year. A total of 20 photos/videos were used for the analysis in this study. Since Instagram is primarily a photo and video sharing application, the ESC assessed fell within these two categories, static image posts and dynamic experimental videos. Analytical data was collected from each post 2 weeks after the content was posted. Data included number of likes, comments, shares, saves, and age demographics of viewers.

### Measurements

To account for the fact that not every post will be seen by the same number of users, we normalized all of the analytical data collected to account for this difference. Both Instagram and TikTok report the “reach” of each individual post, which refers to the total number of unique viewers. As a result, we assess each of the defined analytical outputs as a percentage of total reach. The variables including likes, comments, views, saves, and shares are all expressed as a percentage of total reach. To assess whether the difference in user engagement observed is statistically significant, we performed a paired T-Test (p<0.05).

## Results

### Frequency of engagement

**Fig 1:**
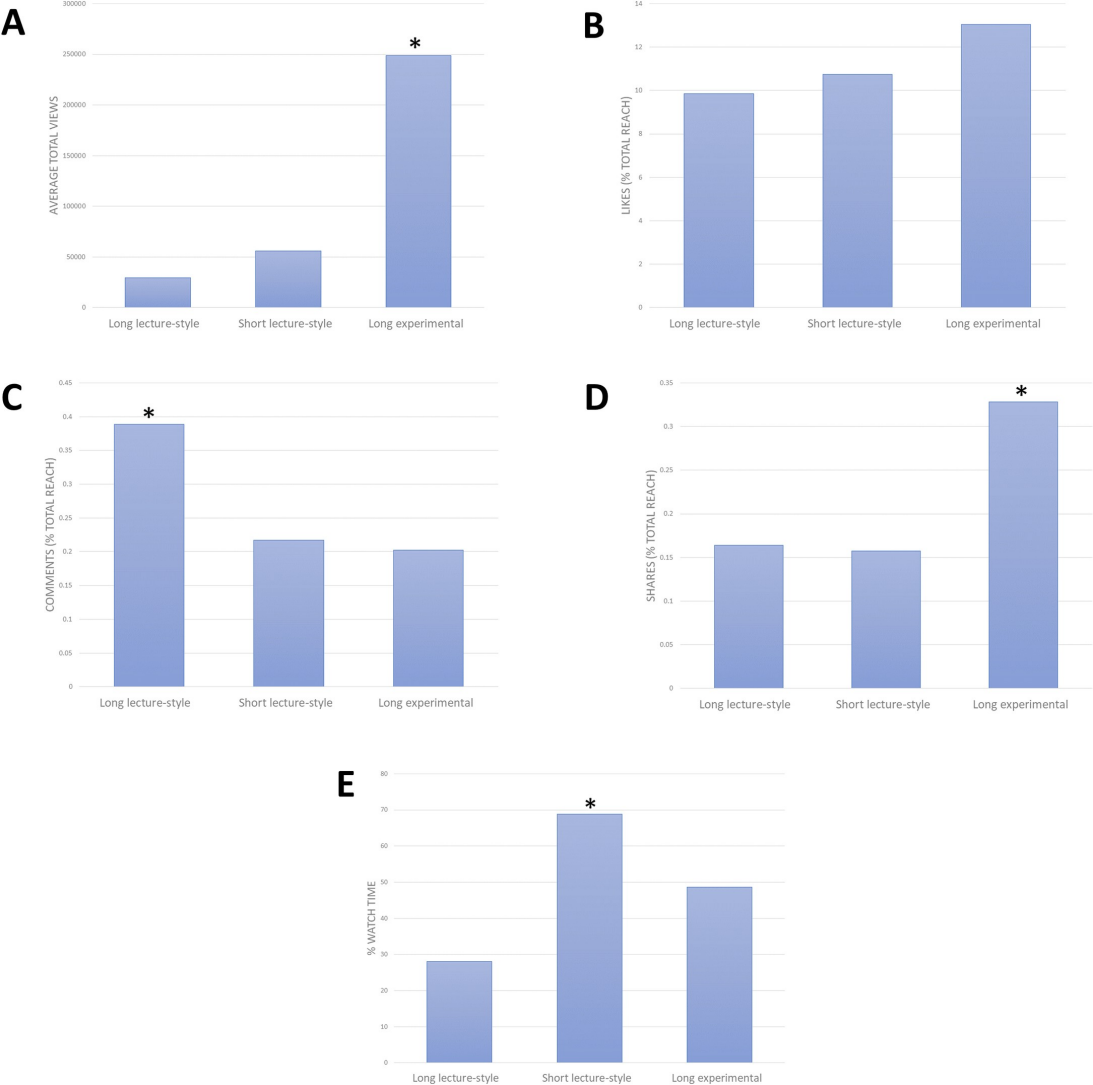
Analytical metric data obtained for three types of ESC videos posted to TikTok platform. The three categories of ESC are long-lecture style videos (40–60 seconds in length), short lecture style videos (15 seconds in length), and long experimental-style videos (40–60 seconds in length). * Indicated statistical significance (p<0.05). **A)** Average total number of views for the three categories of videos. **B)** Average number of likes as a percentage of total post reach for the three categories of videos. **C)** Average number of comments as a percentage of total post reach for the three categories of videos. **D)** Average number of shares as a percentage of total reach for the three categories of videos. **E)** Average percentage watch time for each of the three categories of videos.

The 40 ESC videos that were posted throughout the Creative Learning Fund on TikTok received over 4 million views collectively. The long lecture-style, short lecture-style, and long experimental videos received a total of 260 thousand, 560 thousand, and 3.2 million views, respectively. The average total views for the three categories of videos were 29 thousand, 56 thousand, and 250 thousand, respectively (Fig 1A). The average number of views for the experimental videos is significantly higher than both the long lecture-style (p = 0.01) and short lecture-style videos (p = 0.02). The likes, comments, and shares for each post are represented by a percentage of total post reach (Fig 1B–1D). The long lecture-style, short lecture-style, and long experimental videos received a total of 20 thousand, 72 thousand, and 493 thousand likes, respectively. The average percentage of likes/reach for the long experimental videos was 13%, whereas the long and short lecture-style videos received 9% and 10% likes respectively (Fig 1B). The long lecture-style, short lecture-style, and long experimental videos received a total of 640, 1137, and 3968 comments, respectively. The average percentage of comments/reach was significantly higher for the long lecture-style videos at 0.4%, compared to 0.2% for both short lecture-style (p = 0.02) and long experimental (p = 0.03) videos (Fig 1C). The long lecture-style, short lecture-style, and long experimental videos had a total of 312, 1209, and 10381 shares, respectively. The average percentage of video shares/reach was higher and nearing significance for the long experimental videos at 0.3%, compared to the long (p = 0.07) and short (p = 0.06) lecture-style videos at 16% and 15% respectively (Fig 1D). The average percentage watch time of each video was also assessed (Fig 1E). The average percentage watch time was significantly higher for the short lecture-style video at 69%, compared to 48% for the long lecture-style videos (p = 4.5E-06), and 28% for the long experimental videos (p = 0.003). A summary of the reported data and statistical significance can be observed in Table 1.

**Table 1:** Summary of data collected from the three styles of ESC posted to Instagram and TikTok.

Platform	Type of ESC	Average total views	Likes as a percentage of total reach (%)	Comments as a percentage of total reach (%)	Shares as a percentage of total reach (%)	Percent average watch time (%)	Saves as a percentage of total reach (%)
TikTok	**Long lecture-style**	29576	9.86	0.39*	0.16	28.10	N.A
	**Short lecture-style**	55914	10.74	0.22	0.16	68.85*	N.A
	**Long experimental**	248819*	13.04	0.20	0.33*	48.62	N.A
Instagram	**Static**	14834	7.56	0.32	0.24	N.A	0.36
	**Dynamic**	14598	9.04	0.72	2.27*	N.A	1.87*

* indicates value is significantly higher than the other values presented in the same grouping.

### Dynamic vs. static methods of delivery

**Fig 2:**
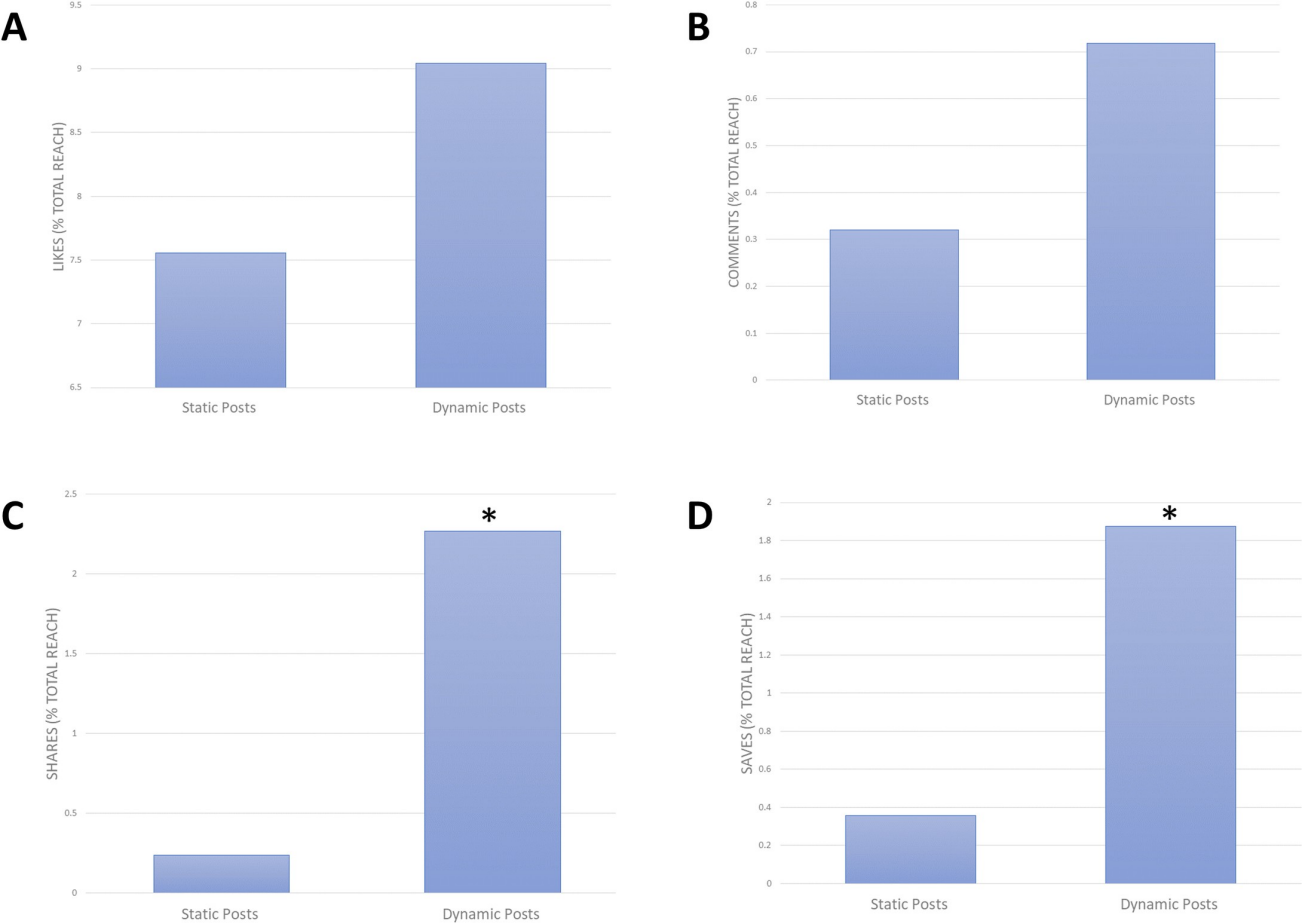
Analytical metric data obtained for two types of ESC videos posted to the Instagram platform. The two categories of ESC are static image posts and long experimental-style videos (40–60 seconds in length). * Indicated statistical significance (p<0.05). **A)** Average number of likes as a percentage of total post reach for the two categories of posts. **B)** Average number of comments as a percentage of total post reach for the two categories of posts. **C)** Average number of shares as a percentage of total post reach for the two categories of posts. **D)** Average number of saves as a percentage of total post reach for the two categories of posts.

The 20 ESC photos and videos posted to Instagram for the duration of this study reached over 200 thousand individuals. The static image posts and dynamic experimental videos received a total of 11 thousand and 14 thousand likes respectively. The likes, comments, shares, and saves for each post are represented by a percentage of total post reach (Fig 2A–2D). The average percentage of likes/reach for the static image posts and dynamic experimental videos were 7% and 9%, respectively (Fig 2A). The static image posts and dynamic experimental videos received a total of 468 and 1177 comments, respectively. The percentage of average number of comments/reach was higher and nearing significance (p = 0.08) for dynamic experimental videos at 0.7%, compared to static image posts at 0.3% (Fig 2B). The two groups of posts received a total of 310 and 3823 shares. The percentage of average number of shares/reach was significantly higher (p = 0.02) for the dynamic experimental videos at 2.7%, compared to static image posts at 0.2% (Fig 2C). The static image posts and dynamic experimental videos were saved a total of 533 and 2922 times. The percentage of average number of post saves was significantly higher (p = 0.002) for the dynamic experimental videos at 1.9%, compared to static image posts 0.4% (Fig 2D). A summary of the reported data and statistical significance can be observed in Table 1.

### Comparison between different social media platforms

**Fig 3:**
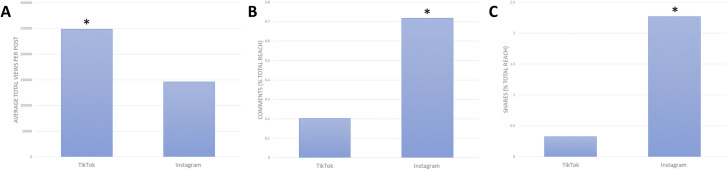
Analytical metric data obtained for similar types of ESC videos posted to the Instagram and TikTok platforms. The similar ESC videos used for comparison purposes between the two platforms were both long experimental-style videos (40–60 seconds in length). * Indicated statistical significance (p<0.05). **A)** Average number of video views for ESC posted on TikTok and Instagram. **B)** Average number of comments as a percentage of total post reach for ESC posted on TikTok and Instagram. **C)** Average number of shares as a percentage of total post reach for ESC posted on TikTok and Instagram.

To compare the two different social media platforms, Instagram and TikTok, in relation to engagement, we assessed the average number of views for the ESC posted to each platform. Since the ESC videos performed significantly better than the static image posts on Instagram, we focused our comparison study on only the videos posted to each platform (Fig 3). The ESC video posts on Instagram received an average of 15 thousand views per post. Whereas the similar videos posted to TikTok received an average of 248 thousand views per post (Fig 3A). This difference observed is statistically significant (p = 0.008). Similarly, the ESC videos on Instagram received an average of 117 comments and 382 shares per post, whereas the similar videos on TikTok received an average of 305 comments and 798 shares per post (Fig 3B and 3C). Although this difference appears large, when we account for the average number of comments and shares relative to the posts total reach, we see Instagram receives 0.7% and 2.7% respectively, and TikTok receives 0.2% and 0.3% respectively. The average number of relative comments (p = 0.00005) and shares (p = 0.003) Instagram received is significantly higher than what was seen on TikTok. A summary of the reported data and statistical significance can be observed in Table 2.

**Table 2:** Summary of data collected comparing the same style of ESC posted to Instagram and TikTok.

Platform	Average total views	Comments as a percentage of total reach (%)	Shares as a percentage of total reach (%)
TikTok	248819*	0.20	0.33
Instagram	145989	0.72*	2.27*

* indicates value is significantly higher than the other values presented in the same grouping.

## Discussion

This study aims to assess the level of user engagement with science communication in the form of educational science content (ESC) on social media. We made use of the analytical data collected from posts made on Instagram and TikTok and analyzed user engagement in terms of likes, views, comments, shares, and saves. The findings of our study address the type of ESC and methods of science communication on social media that stimulate various levels of engagement. We also assess the differences in total user engagement with science across the two online platforms. In order for science communication to be effective, it must have clear and definitive aims and goals [7]. Therefore, here we present suggestions for the best formats of ESC used for science communication on social media depending on the goals of the science communicator.

### Methods of science communication that result in the highest level of engagement on social media

The three types of ESC assessed on TikTok were long lecture-style videos, short lecture-style videos, and long experimental videos. Long lecture-style videos include those that aim to teach a specific concept in science, using primarily speech, for the duration of 40–60 seconds. Short lecture-style videos include those that specifically teach a scientific concept, but present the information in the form of quick facts that are 15 seconds in length. Long experimental videos include those that use hands on demonstrations and a series of experimental steps that are 40–60 seconds in length. We found that the long experimental-style videos on average received the highest number of views per post. Since this difference is significantly higher than the long and short lecture-style videos, we suggest that when communicating science on TikTok, if the goal is to reach the largest audience, the use of experimental style videos has the highest likelihood to do so. That being said, sheer number of views is not always indicative of full engagement. Something also worth noting is the retention rate of the users engaging with the ESC presented on TikTok. In order to assess this, we utilized the “watch time” analytic on TikTok and presented it as a percentage of the video length to highlight the percent watch time of each type of ESC video. It was found that short lecture-style video had a significantly higher percent watch time compared to both of the longer lecture-style and experimental videos. This could suggest that the audience on TikTok has a shorter attention span and thus prefer videos of shorter length. With TikTok being a social platform people turn to in order to get information at accelerated rates, the idea of short videos appealing to larger audiences is in line with that notion. Together, we include a suggestion that if the science communicator wishes to have an increased chance of their science content being viewed completely, they should keep the videos under 15 seconds. That being said, this suggestion should be taken with a piece of caution, as our results did not assess the impact or significance of short experimental style videos. Also, there is little known about whether the platform algorithms influence sorter or longer videos being shown to more or less users.

In order to engage the public with science, science communicators must communicate the scientific information in a way that promotes the users to consult and participate in successive discussions about the topic [29]. Consultation with the ESC presented can be achieved when users provide feedback and ask questions in the comment section of the posts. When assessing the relative number of comments as a percentage of total post reach, we found that long lecture-style videos received the highest number of comments. Thus, we suggest that if the goal of the science communicator is to stimulate discourse and discussion surrounding a topic in science, the format of 40–60 second long lecture-style video is the best fit.

When assessing the relative number of likes and shares as a percentage of total post reach, we found that experimental style videos receive the highest number of likes and shares, when compared to long and short lecture-style videos. Based on the understanding that individuals showing interest in scientific information plays a significant role in sustaining engagement [31], we conclude that long experimental style videos aid in fostering this initial level of engagement. Similarly, as defined in the terms of the current study, we suggest that the highest level of engagement is achieved when users share the information they receive. Some argue that the goal of science communication should be to foster critical engagement rather than blind devotion [32]. Rather than simply acknowledging and liking a piece of ESC, users can immerse deeper into private and individual scientific discussions with peers when they share the information. Thus, we found that if the goal of the science communicator is to foster critical engagement in the form of individualized conversations apart from the initial introduction, the ESC should be presented in the form of experimental videos.

With Instagram being primarily a photo sharing application, the two types of ESC shared were either static image posts, or dynamic experimental videos. The static image posts were primarily self-portraits of researchers in a laboratory setting, whereas the dynamic experimental videos were primarily fast-paced science experiments taking place outside of the laboratory setting. Both types of posts were accompanied by 200–400 word captions, which further described the specific science related topic. When assessing the relative number of likes, comments, shares, and saves as a percentage of total post reach, we found that dynamic experimental videos received the highest level of engagement for all of the variables assessed. With Instagram introducing new in-application video components, such as IGTV (a feed that allows users to post videos that are up to 10 minutes in length) and IG Reels (a feed that resembles the style of videos on TikTok), it is apparent that users enjoy dynamic video posts. Unfortunately, up until now much of the science communication on Instagram is in the form of static images. With our results indicating that dynamic experimental videos result in the highest amount of user acceptance, discussion, and overall engagement, we suggest that science communicators should take advantage of in-app video format options for sharing science on social media.

The final aim of this study was to compare the level of user engagement as a whole between the two social media platforms. We found that overall, the average number of views received on individual experimental videos were significantly higher when the video was posted to TikTok versus Instagram. With prior research indicating that hands-on experimentation with science is one of the most effective ways for students to learn and engage with scientific information in the classroom [5, 24, 25], we hypothesized that this format of teaching would also be most effective on social media. Our results show that although dynamic experimental videos reach the largest audience when posted to TikTok, the relative number of comments and shares, as a ratio of total post reach, is higher when the videos are posted to Instagram. Thus, we suggest that if the goal of the science communicator is to reach the largest audience with their ESC, they should use TikTok. Whereas, if the goal of the science communicator is to promote sharing and conversations among a higher percentage of viewers, they should use Instagram. With research indicating that classroom teaching that incorporates hands-on science experimentation into lessons supports more students to have a positive attitude towards science [27], we aimed to assess this attitude when the experiments were posted online. Although the two social media platforms, TikTok and Instagram, result in different specific outcomes when it comes to user engagement, we still see that engagement, as a whole is highest when ESC is presented in dynamic experimental ways.

### Limitations

A few limitations should be considered when interpreting the results presented in this study. First, the ESC used for the completion of this study was posted to already established social media platforms, rather than being posted to new profiles. The Instagram profile used for this study has been actively taking part in science communication for the past 2 years, whereas the TikTok profile has been actively used for 5 months prior to this study. Similarly, the data collected from TikTok took place during the 2020 global pandemic, and as a result could be a reason why the platform experienced a high volume of users, and successive views and engagement. We aimed to eliminate this factor as much as possible by expressing all of the analytical data as a percentage of total post reach. That way we only compared the relative level of user engagement with ESC, and took into account that not every post will be seen by the same number of individuals and so the resulting analytical data collected will also vary.

Another limitation of possible concern is the fact that both the static posts and dynamic videos posted to Instagram contained a caption describing the content. Some would argue that a better comparison would only have a caption included in the static post, since the dynamic video already explains its content in the video. The concern here would be whether the outcomes we saw of increased user engagement with dynamic videos compared to static posts be altered if the videos no longer included a caption. We feel that since the caption on the dynamic videos acted primarily as descriptive scripts for what was taking place in the videos, there would not be any significant difference in the results obtained if the captions were to be removed.

Finally, we would like to bring awareness to the fact that the two social media platforms used in this study, TikTok and Instagram, function in different manners in regards to who sees content posted to them. On both platforms, users have the option to follow the content creator, and as a result will likely see the majority of the content that creator posts. The key difference in the platforms is in how the users receive the information presented. On Instagram, the primary location for users to receive and view content is through their Instagram Feed. A users Instagram feed is composed of content posted from all of the profiles (content creators) they follow. If users wish to view popular content from profiles they do not follow, and seek to view additional content related to the content they typically like (based on algorithmic suggestions), they can view the Explore feed on Instagram. However, on TikTok the primary location for users to receive and view content is through their “For You” page. Unlike Instagram, the For You page is composed of popular and unpopular content posted from all users on the platform, in addition to the content from the specific profiles they follow. The content present on each individual users For You page uses algorithms to determine content related to the users interests (TikTok Product, 2020). For example, if an individual user engages most with cooking related content, they will primarily see cooking content on their For You page. Thus, in the case of our study the broad distribution of the ESC on TikTok could be a reason why we saw a larger number of view per individual post. With this in mind, since TikTok incorporates users interests into the content users receive, and Instagram only presents content to users from pages they follow, we see in both cases the content presented to the users is influenced by the interest of the user. Since we cannot alter how each social platform functions, nor would it typically be necessary for this type for study, we see this limitation as inevitable. Together, the premise of these limitations lays primarily on the apparent function of each social media platform.

## Conclusion

The presented study assessed various methods of SciComm on two social media platforms, and provided suggested for how to maximize user engagement through understanding the importance of the analytical outputs, likes, comments, shares, saves, and views. We defined the importance of each analytical output in relation to user engagement, and created a method for accurately assessing the relative level of engagement of posts made to various social media platforms. To our best knowledge, this is the first study that critically analyzes each of the analytical output on TikTok and Instagram for the assessment of effective science communication. This method can be applied to future studies that aim to understand the best practices for science communication on social media. Future research that assesses a wider range of ESC could be of use moving forward, in hopes to create a more holistic picture of how to effectively engage users into science that is communicated on social media.

## Supporting information

S1 Data (XLSX)
